# Validation of algorithms to identify colorectal cancer patients from administrative claims data of a Japanese hospital

**DOI:** 10.1186/s12913-023-09266-1

**Published:** 2023-03-21

**Authors:** Takahiro Hirano, Makiko Negishi, Yoshiki Kuwatsuru, Masafumi Arai, Ryozo Wakabayashi, Naoko Saito, Ryohei Kuwatsuru

**Affiliations:** 1 Clinical Study Support, Inc., Daiei Bldg., 2F, 1-11-20 Nishiki, Naka-ku, Nagoya, 460-0003 Japan; 2 Real-World Evidence and Data Assessment (READS), Graduate School of Medicine, Juntendo University, Tokyo, Japan; 3 Shin Nippon Biomedical Laboratories, Ltd., Tokyo, Japan; 4 Department of Radiology, School of Medicine, Juntendo University, Tokyo, Japan

**Keywords:** Colorectal cancer, Administrative claims data, Validation, Diagnostic codes, Japan, Positive predictive value

## Abstract

**Background:**

Administrative claims data are a valuable source for clinical studies; however, the use of validated algorithms to identify patients is essential to minimize bias. We evaluated the validity of diagnostic coding algorithms for identifying patients with colorectal cancer from a hospital’s administrative claims data.

**Methods:**

This validation study used administrative claims data from a Japanese university hospital between April 2017 and March 2019. We developed diagnostic coding algorithms, basically based on the International Classification of Disease (ICD) 10th codes of C18–20 and Japanese disease codes, to identify patients with colorectal cancer. For random samples of patients identified using our algorithms, case ascertainment was performed using chart review as the gold standard. The positive predictive value (PPV) was calculated to evaluate the accuracy of the algorithms.

**Results:**

Of 249 random samples of patients identified as having colorectal cancer by our coding algorithms, 215 were confirmed cases, yielding a PPV of 86.3% (95% confidence interval [CI], 81.5–90.1%). When the diagnostic codes were restricted to site-specific (right colon, left colon, transverse colon, or rectum) cancer codes, 94 of the 100 random samples were true cases of colorectal cancer. Consequently, the PPV increased to 94.0% (95% CI, 87.2–97.4%).

**Conclusion:**

Our diagnostic coding algorithms based on ICD-10 codes and Japanese disease codes were highly accurate in detecting patients with colorectal cancer from this hospital’s claims data. The exclusive use of site-specific cancer codes further improved the PPV from 86.3 to 94.0%, suggesting their desirability in identifying these patients more precisely.

**Supplementary Information:**

The online version contains supplementary material available at 10.1186/s12913-023-09266-1.

## Background

Routinely collected health data, such as administrative data and electronic health records, have a high research potential [1] and, therefore, are increasingly being used in medical research [2]. Administrative claims data have several research strengths, such as large sample size, representativeness of routine clinical care, extensive longitudinal data, and data availability at low cost without long delays [3, 4]. Therefore, in Japan, they are increasingly being used to generate real-world evidence in pharmacoepidemiology.

However, such data should be carefully used because they are not primarily generated for research purposes. Improper use of these data introduces enormous bias, which can mislead conclusions [2]. For example, misclassification of outcomes and exposures is a significant challenge [5] because diagnostic or procedure codes in claims data used for patient identification may not necessarily be clinically accurate. Therefore, researchers should measure the accuracy of coding algorithms before using them to minimize bias [2, 6]. In contrast to Western countries [7, 8], such code validation studies are infrequently conducted in Japan [9], and should be more facilitated to enhance the credibility of real-world evidence.

Colorectal cancer is the third most commonly diagnosed cancer, accounting for 10.0% of all cases globally, and the second leading cause of cancer death [10]. Colorectal cancer was the most commonly diagnosed cancer in Japan in 2018, with over 150,000 new cases diagnosed [11], and approximately 50,000 people died of this cancer annually [12]. Early detection by screening is crucial to reduce the clinical burden, and efforts have been made to promote it. In the United States, during a period of increasing rates in screening, the incidence and mortality rates declined [13, 14]. In contrast, Japan, with a lower screening rate, would have room to further reduce the disease burden by promoting screening [15]. Not only does this cancer affect survival but it also affects the physical and psychological quality of life [16, 17] and causes an economic burden [18]. Given the enormous public health impacts, real-world studies to estimate this disease’s clinical and economic burden are essential.

Recently, in Japan, colorectal cancer research has been conducted using claims data [19–21]; however, the accuracy of the algorithms used has not been reported. Outside Japan, some studies have validated algorithms to identify colorectal cancers [22–24]. However, in Japan, no validation studies specifically targeting colorectal cancer have been conducted, although a few studies targeting all types of carcinomas have suggested the utility of diagnostic codes for identifying cancers in general [25, 26]. Thus, a coding validation study targeting colorectal cancer should be conducted to obtain a valid coding algorithm for identifying patients with this disease from claims data in Japan.

Therefore, this study evaluated the validity of coding algorithms to identify patients with colorectal cancers from a hospital’s administrative claims data in Japan. This validation study assessed the two algorithms based on the International Statistical Classification of Diseases and Related Health Problems, 10th revision (ICD-10) codes and Japanese disease codes, using electronic medical record review as the gold standard for case ascertainment. We evaluated the performance of the algorithms using positive predictive value (PPV) aiming to obtain algorithms to identify patients with colorectal cancer and to examine the treatment effects or the clinical/economic burden.

## Methods

### Data source and population

A cross-sectional study was conducted at Juntendo University Hospital (Tokyo, Japan), a designated regional cancer care hospital with 1,051 beds. We used administrative claims data from the hospital between April 2017 and March 2019. This study used inpatient and outpatient general claims data from patients aged ≥ 18 years who visited the hospital during the study period and has ICD-10 codes C18 (malignant neoplasm of the colon), C19 (malignant neoplasm of the rectosigmoid junction), or C20 (malignant neoplasm of the rectum). For patients included in the analysis, electronic medical records were reviewed as the gold standard for colorectal cancer diagnosis.

This validation study was approved by the Ethics Committee of Juntendo University Hospital. According to the Japanese Ethical Guidelines for Medical and Health Research Involving Human Subjects, informed consent was waived for this retrospective chart and claims data review. However, the study information, including the purpose and data use, was posted on the hospital’s website, ensuring that participants had the right to opt out. All methods were performed in accordance with the relevant guidelines and regulations.

### Algorithms to identify colorectal cancer patients

We developed the following algorithms based on expert opinions and a review of the previous literature [24]:

1. Algorithm 1 Presence of at least one diagnostic code for colorectal cancer (ICD-10 codes: C18.x, C19, and C20) during the study period, excluding the following diseases: carcinoid tumor, neuroendocrine tumor G1/G2, neuroendocrine cell carcinoma, neuroendocrine carcinoma G3, mixed adenoneuroendocrine carcinoma, stromal tumor, sarcoma, and melanoma (Supplementary Table S1).
2. Algorithm 2 Presence of at least one diagnostic code for site-specific colon or rectal cancer during the study period. These codes correspond to the diagnostic codes for Algorithm 1, excluding C18.1 and C18.9.

Table 1 lists the names and codes of the diseases used to define each algorithm. The 7-digit Japanese disease codes for the above-defined excluded diseases are provided in Supplementary Table S1. A disease code marked as a suspected diagnosis using a suspicion flag (i.e., a disease’s name that is assigned to a test order and remains a suspected diagnosis until it is confirmed by a doctor) was not considered as evidence of colorectal cancer, and patients without a disease code that was not marked with a suspicion flag were excluded in both Algorithms 1 and 2.

**Table 1:** Diagnostic codes for colorectal cancer

ICD-10 code	Disease name	Japanese disease code	Side of colon	Definitions	
				Algorithm 1	Algorithm 2
**C18 Malignant neoplasm of colon**					
C18.0 (cecum)	Cecal cancer	1534004	Right colon	X	X
	Ileocecal cancer	1534001	Right colon	X	X
C18.1 (appendix)	Appendix cancer	1535002	Unspecified	X	-
	Malignant appendiceal mucocele	8830219	Unspecified	X	-
C18.2 (ascending colon)	Ascending colon cancer	1536002	Right colon	X	X
C18.3 (hepatic flexure)	Hepatic flexure cancer	8831682	Right colon	X	X
C18.4 (transverse colon)	Transverse colon cancer	1531002	Transverse colon	X	X
C18.5 (splenic flexure)	Splenic flexure cancer	8839429	Left colon	X	X
C18.6 (descending colon)	Descending colon cancer	1532002	Left colon	X	X
C18.7 (sigmoid colon)	Sigmoid colon cancer	1533003	Left colon	X	X
C18.9 (colon, unspecified)	Colon cancer	1539002	Unspecified	X	-
	Colorectal cancer	1539004	Unspecified	X	-
	KRAS wild-type colon cancer	8847915	Unspecified	X	-
	Hereditary colorectal cancer	8842670	Unspecified	X	-
	Hereditary nonpolyposis colorectal cancer	8842671	Unspecified	X	-
	Colorectal mucinous carcinoma	8842802	Unspecified	X	-
**C19 Malignant neoplasm of rectosigmoid junction**					
	Malignancy of rectosigmoid junction	8848749	Left colon	X	X
	Rectosigmoid cancer	8850538	Left colon	X	X
**C20 Malignant neoplasm of rectum**					
	KRAS wild-type rectal cancer	8847916	Rectum	X	X
	Rectal cancer	1541005	Rectum	X	X
	Postoperative recurrence of rectal cancer	1541009	Rectum	X	X
	Perforated rectal cancer	1541010	Rectum	X	X

### Sampling

The minimum sample size was set at 100 for each algorithm cohort. This number is considered sufficient to evaluate the coding accuracy in a random sampling of patients meeting the outcome [27].

To reduce the number of cases for chart review of electronic medical records, we planned to sample patients for Algorithm 2 from random samples for Algorithm 1. When we randomly sampled 250 patients for Algorithm 1, it contained more than 100 patients who also met Algorithm 2. Although the number of patients who met Algorithm 2 (102 patients) was almost the same as the aimed sample number (100 patients), we randomly sampled 100 patients for Algorithm 2 from the 102 patients according to our planned procedure. A chart review was performed for all 250 samples.

### Chart review and case ascertainment

Two physicians independently reviewed the patients’ medical records to ascertain the presence of colorectal cancer and the primary tumor location. Data were examined for the first diagnosis month ± 6 months. The first diagnosis month refers to the month when the diagnostic code for the above-defined colorectal cancer first appeared in the patients’ claims data at the hospital.

An accurate diagnosis was ascertained based on documentation of the presence of colorectal cancer in medical records. Similarly, tumor location was ascertained based on such documentation. Additionally, data on the degree of differentiation and cancer stage were retrieved if available. The final diagnosis was made when the two physicians’ judgments agreed. If their decisions disagreed, they discussed the conclusion. If necessary, the evaluators reviewed the data beyond the above-defined period. Patients were excluded from the analysis if they were unable to judge based on a chart review.

### Statistical analyses

The patients’ background characteristics were summarized for each algorithm’s total and sampled patients. The coding accuracy of each algorithm was evaluated using PPV. PPV was calculated as the proportion of confirmed cases based on chart review (gold standard) among all samples (i.e., patients identified as a case by each algorithm). The 95% confidence interval (CI) of the PPV, assuming a binomial distribution, was calculated using Wilson’s method [28]. All statistical analyses were performed using R version 4.1.0 (R Foundation for Statistical Computing, Vienna, Austria).

## Results

### Patients’ background characteristics

**Fig. 1:**
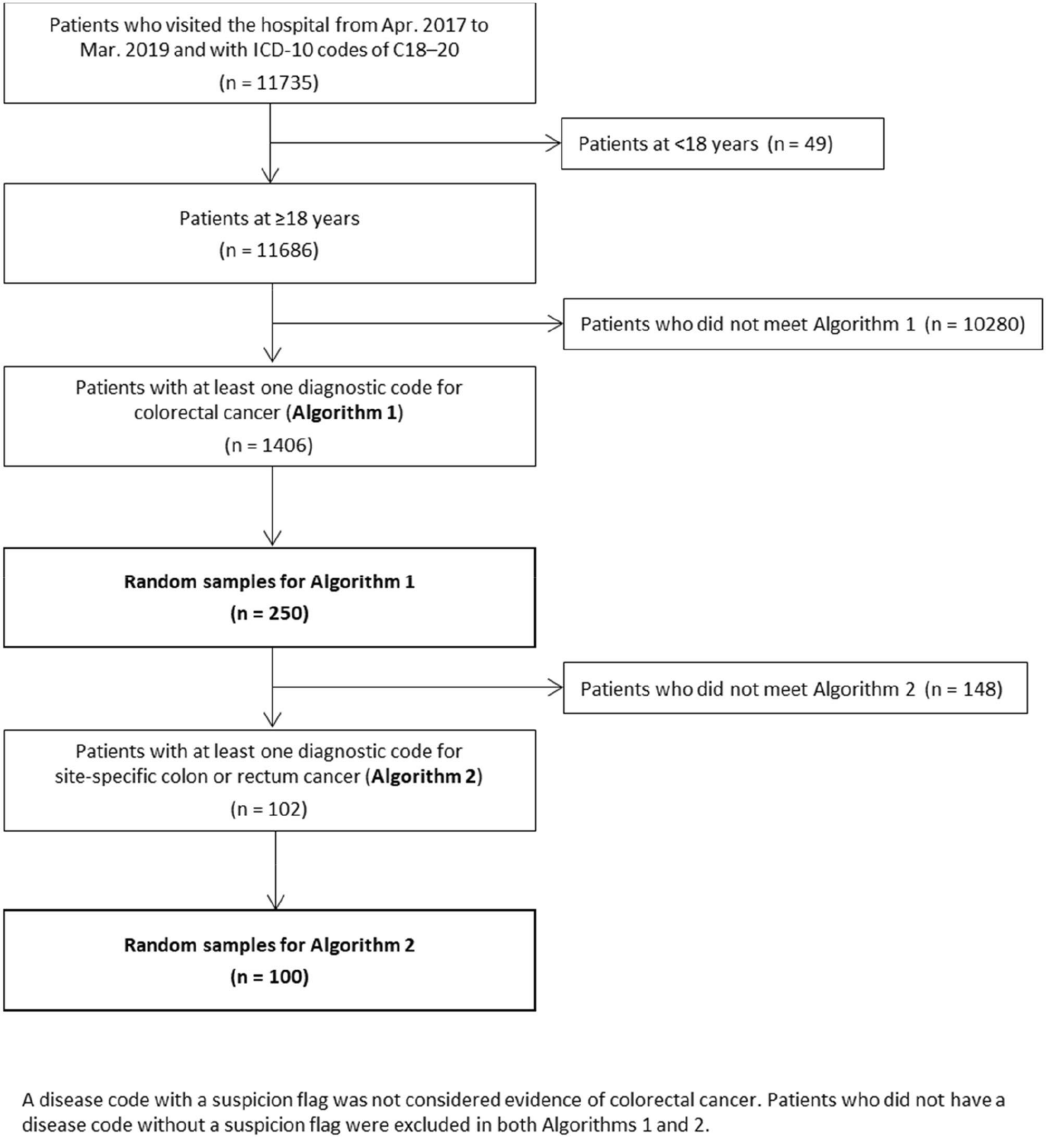
Flow diagram of patient selection

Of the 11,686 patients aged ≥ 18 years, Algorithm 1 identified 1,406 patients with colorectal cancer, from which we randomly selected 250 patients (Fig. 1). Of these, one patient was excluded because the correct diagnosis could not be determined based on chart review. Almost all the excluded patients (10,243 patients out of 10,280 patients) had only suspected diagnosis records.

The demographic characteristics and diagnosis distributions were similar between the total target population and sampled patients (Table 2). The mean ± standard deviation (SD) age of the 249 patients was 66.69 ± 13.82 years, and 54.22% were men. The most common diagnoses were colorectal cancer (33.73%), rectal cancer (22.09%), and sigmoid colon cancer (20.48%). According to chart review, the primary tumor location was identified in 83.13% (n = 207) of patients, with the majority in the rectum (n = 137), followed by the right-sided colon (n = 37), transverse colon (n = 23), and left-sided colon (n = 10).

**Table 2:** Background characteristics of patients identified by Algorithms 1 and 2

Characteristics	Algorithm 1: Colorectal cancer codes					Algorithm 2: Site-specific colorectal cancer codes			
	Total (n = 1,406)		Samples (n = 249)			Total^b^ (n = 524)		Samples (n = 100)	
Age									
Mean ± SD	66.60 ± 13.07		66.69 ± 13.82			67.63 ± 12.57		67.83 ± 13.32	
Median [min–max]	69.0 [21–96]		69.0 [21–95]			69.0 [21–96]		69.5 [21–90]	
Men, n (%)	815	(57.97)	135	(54.22)		285	(54.39)	50	(50.00)
Diagnosis, n (%)^a^									
Colorectal cancer	512	(36.42)	84	(33.73)		15	(2.86)	2	(2.00)
Rectal cancer	348	(24.75)	55	(22.09)		6	(1.15)	–	–
Sigmoid colon cancer	226	(16.07)	51	(20.48)		226	(43.13)	51	(51.00)
Ascending colon cancer	141	(10.03)	21	(8.43)		141	(26.91)	21	(21.00)
Transverse colon cancer	76	(5.41)	16	(6.43)		76	(14.5)	14	(14.00)
Cecal cancer	56	(3.98)	10	(4.02)		56	(10.69)	10	(10.00)
Descending colon cancer	33	(2.35)	7	(2.81)		33	(6.3)	7	(7.00)
Colon cancer	18	(1.28)	6	(2.41)		–	–	–	–
Postoperative recurrence of rectal cancer	16	(1.14)	4	(1.61)		–	–	–	–
Appendix cancer	15	(1.07)	–	–		–	–	–	–
Ileocecal cancer	–	–	–	–		6	(1.15)	2	(2.00)

^a^The earliest diagnostic records for colorectal cancer are tabulated. Some patients had multiple diagnoses on the same day. Only diagnoses with a proportion of > 1.0% were listed

^b^Data of 524 patients who met Algorithm 2 within all records obtained are shown instead of 102 patients who met Algorithm 2 within the sampled patients for Algorithm 1

Note: SD, standard deviation

Of the 102 patients who met Algorithm 2, we randomly selected 100 patients (Fig. 1). The mean ± SD age was 67.83 ± 13.32 years, and half were men (Table 2). The top three most common diagnoses were sigmoid colon cancer (51.00%), ascending colon cancer (21.00%), and transverse colon cancer (14.00%). According to chart review, the most common primary tumor site was the rectum (n = 50), followed by the right-sided colon (n = 28), transverse colon (n = 10), and left-sided colon (n = 6).

No notable differences existed in patient demographics and cancer characteristics (e.g., degree of differentiation and cancer stage) between the samples using Algorithms 1 and 2 (Tables 2 and 3).

**Table 3:** Primary tumor location and degree of differentiation of patients identified by chart review

Characteristics	Algorithm 1: Colorectal cancer codes			Algorithm 2: Site-specific colorectal cancer codes	
	Samples (n = 249)			Samples (n = 100)	
Primary tumor location, n (%)					
Left-sided colon	10	(4.02)		6	(6.00)
Right-sided colon	37	(14.86)		28	(28.00)
Transverse colon	23	(9.24)		10	(10.00)
Rectum	137	(55.02)		50	(50.00)
Unknown	42	(16.87)		6	(6.00)
Degree of differentiation, n (%)					
Poorly differentiated	6	(2.41)		3	(3.00)
Moderately differentiated	89	(35.74)		37	(37.00)
Well-differentiated	57	(22.89)		27	(27.00)
Unknown	97	(38.96)		33	(33.00)
Cancer stage, n (%)					
0	12	(4.82)		4	(4.00)
I	44	(17.67)		18	(18.00)
II	37	(14.86)		21	(21.00)
III	32	(12.85)		12	(12.00)
IV	53	(21.29)		25	(25.00)
Unknown	71	(28.51)		20	(20.00)

### Accuracy of the diagnostic coding algorithms

For Algorithm 1, 215 of 249 patients were confirmed to have colorectal cancer, resulting in a PPV of 86.3% (95% CI, 81.5–90.1%) (Table 4). In Algorithm 2, 94 of the 100 patients were accurate colorectal cancer patients. Thus, the PPV was 94.0% (95% CI, 87.2–97.4%). Restricting codes to the use of site-specific cancer codes improved the PPV by 7.7% (from 86.3 to 94.0%).

**Table 4:** Positive predictive values of diagnostic coding algorithms to identify patients with colorectal cancer

Algorithms	Potential cases^a^	Confirmed case by chart review	PPV (%, 95% CI)
1) Colorectal cancer codes	249	215	86.3 (81.5 to 90.1)
2) Site-specific colon or rectal cancer codes	100	94	94.0 (87.2 to 97.4)

^a^Patients identified by each coding algorithm

PPV, positive predictive value; CI, confidence interval

## Discussion

In this study, we evaluated the accuracy of claims-based coding algorithms for identifying patients with colorectal cancer using administrative claims data from a Japanese hospital. Our diagnostic coding algorithm for colorectal cancer, based on ICD-10 codes C18–C20 with detailed definitions by Japanese disease codes, had a high PPV of 86.3%. Furthermore, the exclusive use of site-specific cancer codes identified the target population more accurately with an excellent PPV of 94.0%.

In Japan, some studies have previously evaluated the coding algorithms for identifying various carcinomas from administrative databases [25, 26, 29]. One study reported that the algorithm based on diagnostic codes plus imaging records had a PPV of 80.8% to identify colorectal cancer in a small subanalysis (n = 28) [25]. Another study, which used the cancer registry data as the gold standard, showed that the algorithm based on diagnostic codes plus treatment conditions (chemotherapy/radiation/surgery) had a PPV of 84.4% to detect this cancer [26]. Direct comparison of our results with these previous results is meaningless because of different databases and methods. However, our algorithm, which used only diagnostic codes, had a higher PPV of 86.3%. This favorable result may be partly because we excluded several diseases instead of using ICD-10 codes directly, suggesting that the diagnostic codes may serve sufficiently without extra procedural conditions if refined and closely defined.

Similar to our results, a few studies have also reported the utility of diagnostic codes alone for identifying colorectal cancer from administrative databases. For example, an Italian study reported that the ICD-9 code-based algorithm had a PPV of 80–81% for colon cancer and 80–84% for rectal cancer, depending on databases [23]. A single-center Korean study reported an excellent PPV of 99.68%. This high accuracy was in part because they used Korean-specific V codes in addition to the ICD-10 codes [22]. Furthermore, in the United States, Luhn et al. documented that the side-specific ICD-9/10 codes for colon cancer were useful in identifying tumor location, with a PPV of 64–92% depending on tumor location [24].

In this study, restricting codes to site-specific cancer codes further increased PPV by 7.7%, resulting in a PPV of 94.0%. A previous study reported a similar trend, showing that the concordance between the ICD-9/10 codes and true diagnosis abstracted from electronic medical records improved after restricting patients to those with site-specific colon cancer codes [24]. A site-specific code is likely to be assigned after diagnosis is confirmed through, for example, a pathological examination, which probably increases the accuracy of site-specific diagnostic codes. It is known that clinical and molecular characteristics differ between the sides of the colon [30], with right-sided cancers being associated with worse prognoses than left-sided [31–33]. Primary tumor location may additionally predict treatment outcomes [34–36]. Given these, the use of site-specific codes, which allows patient stratification, will contribute to real-world studies of this cancer.

Among the various measures for diagnostic accuracy, a high PPV is essential for identifying patients with a target disease especially in comparative studies because, with high PPVs, it is expected that the non-differential sensitivity of disease misclassifications would not bias the risk ratio between groups of interest [27, 37]. Therefore, we focused on PPV in this study and prioritized maximizing the inclusion of true cases while minimizing false cases. However, an algorithm with a high PPV can have high specificity, potentially sacrificing its sensitivity [37]. Thus, it should be noted that our algorithms may not be appropriate for estimating the incidence or prevalence of colorectal cancer.

Currently, commercially available Japanese claims databases are not linked to patient medical records because of strict restrictions on data linkage, which poses an obstacle to validation studies in Japan. Therefore, this study used claims data from a hospital, which we compared with electronic medical records. The use of chart review as the gold standard is one of the major strengths of this validation study because it is considered the most reliable. Another strength is its high internal validity. The demographic characteristics of our samples were similar to those of the overall target population, indicating that they were highly representative of the target population.

This study had several limitations. First, the generalizability of the results was limited because this validation study was conducted using claims data from a single hospital. Therefore, our results may not apply to settings where patients, administrative procedures, or diagnostic practices differ from ours. Second, the prevalence of colorectal cancer might be higher in this university hospital than in others because patients are more likely to be referred to such a hospital capable of providing advanced care for a confirmed diagnosis or undergoing surgeries. This potentially high prevalence likely contributed to the high PPVs in this study. Third, we did not evaluate the accuracy of site-specific codes in identifying tumor location, which should be further validated in a future study. Fourth, we did not evaluate the sensitivity of the algorithms. We intended to use the algorithms in comparative studies to examine treatment effects or clinical/economic burdens, but not in studies to estimate the incidence or prevalence. In such studies, further characterization of the algorithms, sensitivity and specificity, would be necessary. Despite these limitations, we believe that the results of this study will be informative for researchers, providing valuable evidence on the utility of diagnostic codes to detect patients with colorectal cancer from administrative claims data.

## Conclusion

In conclusion, this validation study demonstrated that the diagnostic coding algorithms based on ICD-10 codes and Japanese disease codes had high accuracy in detecting colorectal cancer patients from a hospital’s claims data in Japan. Furthermore, exclusively using site-specific codes for colon or rectal cancer improved the PPV from 86.3 to 94.0%, indicating that site-specific codes are more precise for detecting patients with colorectal cancer. Although generalizability is limited, we hope that our results will be helpful for future real-world studies of this critical clinical condition in Japan.

## Electronic supplementary material

Below is the link to the electronic supplementary material.

Supplementary Material 1

## Data Availability

The dataset generated during the current study is available from the corresponding author on reasonable request.

## List of abbreviations

ICD-10: International Statistical Classification of Diseases and Related Health Problems 10th revision
PPV: positive predictive value
CI: confidence interval
SD: standard deviation
