# Supplementary material for: Validation of algorithms to identify colorectal cancer patients from administrative claims data of a Japanese hospital
Source: BMC Health Serv Res. 2023 Mar 21;23:274. doi: 10.1186/s12913-023-09266-1 (PMC10029250; doi:10.1186/s12913-023-09266-1)
Supplement: Supplementary file 1 — Supplementary Material 1 [file 12913_2023_9266_MOESM1_ESM.docx]

**Supplementary Table S1. Diseases excluded from the algorithms for identifying colorectal cancer**

| **ICD-10 code** | **Disease Name** | **Japanese**  **disease codes** |
| --- | --- | --- |
| **C18: Malignant neoplasm of colon** | |  |
| C18.0 | Cecal carcinoid | 8844619 |
| C18.0 | Neuroendocrine carcinoma of cecum | 8850592 |
| C18.0 | Neuroendocrine cell carcinoma of cecum | 8849216 |
| C18.0 | Neuroendocrine tumor of cecum | 8849217 |
| C18.1 | Appendix carcinoid | 8844547 |
| C18.1 | Appendix goblet cell carcinoid | 8847052 |
| C18.1 | Neuroendocrine carcinoma of appendix | 8850534 |
| C18.1 | Neuroendocrine tumor of appendix | 8849175 |
| C18.2 | Ascending colon carcinoid | 8844478 |
| C18.2 | Neuroendocrine carcinoma of ascending colon | 8850466 |
| C18.2 | Neuroendocrine cell carcinoma of ascending colon | 8849137 |
| C18.2 | Neuroendocrine tumor of ascending colon | 8849138 |
| C18.2 | Leiomyosarcoma of ascending colon | 8835410 |
| C18.4 | Neuroendocrine carcinoma of transverse colon | 8850387 |
| C18.4 | Neuroendocrine cell carcinoma of transverse colon | 8849076 |
| C18.4 | Neuroendocrine tumor of transverse colon | 8849077 |
| C18.6 | Neuroendocrine carcinoma of descending colon | 8850394 |
| C18.6 | Neuroendocrine cell carcinoma of descending colon | 8849082 |
| C18.6 | Neuroendocrine tumor of descending colon | 8849083 |
| C18.7 | Neuroendocrine carcinoma of sigmoid colon | 8850368 |
| C18.7 | Neuroendocrine cell carcinoma of sigmoid colon | 8849061 |
| C18.7 | Neuroendocrine tumor of sigmoid colon | 8849062 |
| C18.9 | Liposarcoma of colon | 8844962 |
| C18.9 | Colonic gastrointestinal stromal tumor | 8847856 |
| C18.9 | Neuroendocrine carcinoma of colon | 8850414 |
| C18.9 | Neuroendocrine cell carcinoma of colon | 8849104 |
| C18.9 | Neuroendocrine tumor of colon | 8849105 |
| C18.9 | KIT (CD117)-positive colonic gastrointestinal stromal tumor | 8847828 |
| C18.9 | Colorectal carcinoid | 8837365 |
| C18.9 | Neuroendocrine carcinoma of large intestine | 8850528 |
| C18.9 | Neuroendocrine cell carcinoma of large intestine | 8849167 |
| C18.9 | Neuroendocrine tumor of large intestine | 8849168 |
| C18.9 | Colorectal sarcoma | 8837377 |
| **C20: Malignant neoplasm of rectum** | |  |
| C20 | KIT (CD117)-positive rectal gastrointestinal stromal tumor | 8847831 |
| C20 | Malignant melanoma of rectum | 8837776 |
| C20 | Rectal carcinoid | 8837779 |
| C20 | Liposarcoma of rectum | 8845017 |
| C20 | Rectal gastrointestinal stromal tumor | 8847886 |
| C20 | Neuroendocrine carcinoma of rectum | 8850539 |
| C20 | Neuroendocrine cell carcinoma of rectum | 8849176 |
| C20 | Neuroendocrine tumor of rectum | 8849177 |
| C20 | Leiomyosarcoma of rectum | 8846018 |
